# Identification of Nitrogen Consumption Genetic Variants in Yeast Through QTL Mapping and Bulk Segregant RNA-Seq Analyses

**DOI:** 10.1534/g3.117.042127

**Published:** 2017-06-05

**Authors:** Francisco A. Cubillos, Claire Brice, Jennifer Molinet, Sebastién Tisné, Valentina Abarca, Sebastián M. Tapia, Christian Oporto, Verónica García, Gianni Liti, Claudio Martínez

**Affiliations:** * Centro de Estudios en Ciencia y Tecnología de Alimentos (CECTA), Universidad de Santiago de Chile (USACH), Chile; † Millennium Nucleus for Fungal Integrative and Synthetic Biology (MN-FISB), 9170201 Santiago, Chile; ‡ Departamento de Ciencia y Tecnología de los Alimentos, Universidad de Santiago de Chile (USACH), 9170201 Chile; § Centre de coopération internationale en recherche agronomique pour le développement (CIRAD), UMR AGAP (Genetic Improvement and Adaptation of Mediterranean and Tropical Plant Research Unit), Campus International de Baillarguet, 34398 Montpellier, France; ** Institute for Research on Cancer and Ageing of Nice (IRCAN), Centre national de la recherche scientifique (CNRS) unités mixtes de recherche (UMR) 7284, Institut national de la santé et de la recherche médicale (INSERM) U1081, University of Nice Sophia Antipolis, 06107, France

**Keywords:** nitrogen, yeast, QTL, RNA-seq, BSR-seq, MPP, multiparental populations

## Abstract

*Saccharomyces cerevisiae* is responsible for wine must fermentation. In this process, nitrogen represents a limiting nutrient and its scarcity results in important economic losses for the wine industry. Yeast isolates use different strategies to grow in poor nitrogen environments and their genomic plasticity enables adaptation to multiple habitats through improvements in nitrogen consumption. Here, we used a highly recombinant *S. cerevisiae* multi-parent population (SGRP-4X) derived from the intercross of four parental strains of different origins to identify new genetic variants responsible for nitrogen consumption differences during wine fermentation. Analysis of 165 fully sequenced F12 segregants allowed us to map 26 QTL in narrow intervals for 14 amino acid sources and ammonium, the majority of which represent genomic regions previously unmapped for these traits. To complement this strategy, we performed Bulk segregant RNA-seq (BSR-seq) analysis in segregants exhibiting extremely high and low ammonium consumption levels. This identified several QTL overlapping differentially expressed genes and refined the gene candidate search. Based on these approaches, we were able to validate *ARO1*, *PDC1*, *CPS1*, *ASI2*, *LYP1*, and *ALP1* allelic variants underlying nitrogen consumption differences between strains, providing evidence of many genes with small phenotypic effects. Altogether, these variants significantly shape yeast nitrogen consumption with important implications for evolution, ecological, and quantitative genomics.

Alcoholic fermentation is exclusively carried out by yeasts and the process transforms sugar within the grape must into ethanol and CO_2_. In this context, a large number of studies have exploited a large panel of *Saccharomyces cerevisiae* strains and classified them into two groups: domesticated and wild populations (Querol *et al.* 2003; Legras *et al.* 2007; Sicard and Legras 2011; Cubillos *et al.* 2011). Thorough phylogenetic analysis revealed four evolutionary lineages (Liti *et al.* 2009), with many other lineages recently found (G. Liti and J. Schacherer, personal communication). *S. cerevisiae* strains show the greatest fermentation capacity in wine-making environments (Torija *et al.* 2001; Beltran *et al.* 2002), mostly because of their high tolerance to ethanol (Arroyo-Lopez *et al.* 2010) and production of secondary-end metabolites that positively contribute to wine character. Yeast needs various nutrients to perform these complex biochemical transformations, predominantly nitrogen and carbon sources, which are found in variable concentrations. Nitrogen represents 6–10% of cellular biomass and is primarily used by yeast for growth and protein synthesis through a wide variety of nitrogen-containing compounds. These compounds are present in natural must and the main examples are ammonium ions (NH_4_^+^) and amino acids. The availability of assimilable nitrogen in musts influences the fermentation performance of yeast cells. For example, musts with low nitrogen concentrations are problematic and generally lead to sluggish fermentations (Salmon 1989) and the production of undesirable volatile compounds. Thus, to avoid problematic fermentations due to nitrogen deficiency, winemakers add di-ammonium phosphate to musts to counterbalance this nitrogen deficit. However, in winemaking this practice is not systematic, since poor control of the procedure may cause microbial instability and undesirable effects on the final sensory profile.

Nitrogen sources can have different impacts on cell development depending on their classification as preferred or not-preferred sources. Ammonium, glutamine, glutamate, and asparagine are preferred nitrogen sources whereas urea, proline, and allantion are not-preferred (Magasanik and Kaiser 2002; ter Schure *et al.* 2000). Yeast preferentially uses substrates that allow the best growth through a regulation mechanism called nitrogen catabolite repression (NCR). This system promotes the expression of permeases for the preferred nitrogen source and the degradation of permeases of those not-preferred sources (Magasanik and Kaiser 2002). Thus, during the fermentation process, yeast cells adjust their metabolism depending on the nitrogen sources available (Rossignol *et al.* 2003; Gasch *et al.* 2000), mostly through TOR pathway regulation. Indeed, TOR is able to sense the quantity and quality of available nitrogen sources and, thus, adapt cell metabolism by regulating the activity of certain systems such as NCR (Pedruzzi *et al.* 2003; Swinnen *et al.* 2006).

Previous studies have shown that nitrogen assimilation profiles vary between strains (Gutierrez *et al.* 2013; Crepin *et al.* 2012; Jara *et al.* 2014; Brice *et al.* 2014a; Ibstedt *et al.* 2015). These different phenotypes result from the high genetic diversity that exists between strains involving a large number of allelic variants (Salinas *et al.* 2016). These variants can be dissected through QTL approaches (Cubillos 2016). In *S. cerevisiae*, several QTL analyses have already focused on identifying the genetic bases for specific physiological traits such as cell morphology (Nogami *et al.* 2007), sporulation (Ben-Ari *et al.* 2006; Katou *et al.* 2009), drug sensitivity (Kim and Fay 2007), flocculation (Brauer *et al.* 2006), wine aroma production (Steyer *et al.* 2012), ethanol tolerance, and growth (Katou *et al.* 2009; Nogami *et al.* 2007; Hu *et al.* 2007; Marullo *et al.* 2007; Smith and Kruglyak 2008), together with sulfur assimilation (Noble *et al.* 2015). Because of the importance of nitrogen in fermentation processes, preliminary research has been carried out on nitrogen consumption (Jara *et al.* 2014; Brice *et al.* 2014b; Yang *et al.* 2016). For oenological strains, variation in nitrogen assimilation profiles are explained by polymorphisms in genes involved in mechanisms related to nitrogen signaling (Brice *et al.* 2014b). Our group previously demonstrated that these variations in nitrogen signaling mechanisms can also be observed between strains from different lineages [Wine/European (WE) and Sake (SA)], identifying three genes for which allelic variation resulted in differences in nitrogen assimilation (Jara *et al.* 2014). Two of these genes, *GLT1* and *GDH2* (a glutamate synthetase and a glutamate dehydrogenase, respectively), belong to the central nitrogen metabolism pathway. Nevertheless, this study only involved two different genetic backgrounds and omitted a larger number of strains derived from different origins that may have developed independent adaptive responses to nitrogen assimilation based on the availability of nitrogen compounds in the distinct environments (Cubillos *et al.* 2011; Salinas *et al.* 2012; Tesniere *et al.* 2015).

Although biparental crosses have brought fruitful results, their power is limited to the extent of genetic variation between the two parental strains and identified QTL can only explain a small percentage of the total phenotypic variance within the species. Instead, utilizing an alternative design incorporating multi-parent mapping populations has proven successful for the mapping of larger number of QTL in several popular model organisms, for instance: *Drosophila* (Long *et al.* 2014), mice (Bogue *et al.* 2015; Durrant *et al.* 2011), and plants (Kover *et al.* 2009; Huang *et al.* 2011). Recently, Cubillos *et al.* (2013) used this type of strategy on the yeast model based on a mapping population obtained from outcrossing four founders representative of the main *S. cerevisiae* lineages, denominated SGRP-4X. Given the high genetic diversity and recombination levels in budding yeast, this type of analysis has shown the advantage of breaking linkage disequilibrium and increasing haplotype diversity. Based on these antecedents, we investigated the genetic basis of nitrogen assimilation variation between strains from various origins by performing linkage mapping in SGRP-4X. We have combined our QTL approach with a BSR-seq analysis in segregants exhibiting extremely high and low ammonium consumption levels. Confirmation by RNA mass sequencing and the identification of multiple allelic variants demonstrates that this is a successful strategy for mapping low effect genes.

## Materials and Methods

### Parental strains and SGRP-4X segregants

Haploid parental strains YPS128 (“NA,” *Mat a*, *ho*::*HygMX*, *ura3*::*KanMX*), DBVPG6044 (“WA,” *Mat a*, *ho*::*HygMX*, *ura3*::*KanMX*), DBVPG6765 (“WE,” *Mat a*, *ho*::*HygMX*, *ura3*::*KanMX*), and Y12 (“SA,” *Mat* α *ho*::*HygMX*, *ura3*::*KanMX*), together with the 165 SGPR-4X segregants used in this study, were previously described (Cubillos *et al.* 2009, 2013). Briefly, to generate SGRP-4X segregants, North American (NA) and West African (WA) strains (both *ura3*::*KanMX*) were crossed to generate diploid F1 hybrids as well as SA and WE strains (both *lys2*::*URA3*). To confirm successful crosses, individual colonies were isolated and mating tests using tester strains Y55-2369 (*MATa*, *ho*Δ, *ura2-1*, *tyr1-1*) and Y55-2370 (*MATa*, *ho*Δ, *ura2-1*, *tyr1-1*), as well as diagnostic PCR for the *MAT* locus (Huxley *et al.* 1990), were performed. F1 hybrids were replicated onto KAc at 23° for sporulation for 10 d before cells were collected in water, treated with an equal amount of ether, and vortexed for 10 min to kill unsporulated cells. After washing the cells in water, they were treated with Zymolase (10 mg/ml) to remove asci. Cell mixtures were vortexed for 5 min to increase spore dispersion. Spores from both crosses were mixed, grown on YPD, and replica plated on minimal media to select for successful crosses. This procedure was repeated 11 times to create the F12 population. Viable spores with correct 2:2 segregations for the *MAT* locus, and *ura3* and *lys2* auxotrophies, were selected. We picked a total of 192 segregants (some from the same tetrad), out of which 165 were used in this study. All the strains used in this work were short-term maintained on YPDA solid media (2% glucose, 0.5% peptone, 0.5% yeast extract, and 2% agar). Genotypes are described in Supplemental Material, Table S1 and File S1.

### Fermentation and nitrogen assimilation estimation

Fermentations were carried out as previously described (Jara *et al.* 2014). Briefly, each individual was fermented in duplicate in synthetic wine must (SM300) and prepared according to Rossignol *et al.* (2003). SM300 was supplemented with a final concentration of 300 mgN/L of assimilable nitrogen (YAN) corresponding to 120 mgN/L of ammonium and 180 mgN/L of a mixture of 19 amino acids (612.6 mg/L L-proline, 503.5 mg/L L-glutamine, 503.5 mg/L L-arginine monohydrochloride, 179.3 mg/L L-tryptophan, 145.3 mg/L L-alanine, 120.4 mg/L L-glutamic acid, 78.5 mg/L L-serine, 75.92 mg/L L-threonine, 48.4 mg/L L-leucine, 44.5 mg/L L-aspartic acid, 44.5 mg/L L-valine, 37.9 mg/L L-phenylalanine, 32.7 mg/L L-isoleucine, 50.0 mg/L L-histidine monohydrochloride monohydrate, 31.4 mg/L L-methionine, 18.3 mg/L L-tyrosine, 18.3 mg/L L-glycine, 17.0 mg/L L-lysine monohydrochloride, and 13.1 mg/L L-cysteine). The strains were initially grown under constant agitation in 10 ml of SM300 for 16 hr at 25°. Next, 1 × 10^6^ cells/ml were inoculated into 12 ml of SM300 (in 15 ml conical tubes) and incubated at 25°, with no agitation for 6 d, the stage at which most nitrogen consumption differences can be observed (Martínez *et al.* 2013; Jara *et al.* 2014). After 6 d, 12 ml of synthetic grape must (SM300) were centrifuged at 9000 × *g* for 10 min and the supernatant was collected. 20 μl of SM300 were injected in a Shimadzu Prominence HPLC equipment (Shimadzu) using a Bio-Rad HPX –87H column according to Nissen *et al.* (1997). The concentration of each amino acid was measured using the HPLC analysis as previously described (Gomez-Alonso *et al.* 2007). The consumption of each nitrogen source was estimated as the difference between the initial and final amounts of each source before and after fermentation, respectively. Phenotyping results are described in Table S2.

### QTL mapping

QTL mapping was performed using the linear mixed effect model, with QTL effect as the fixed effect and genetic background controlled with a polygenic random effect as proposed in Bernardo (2013). Independently, on each case for the 15 phenotypes tested, QTL presence was assessed for each of the 99,900 segregating sites identified in Cubillos *et al.* (2013) (see Table S1) in the linear mixed model as follows:

$$Y=X\beta +X_{k}\beta_{k}+Zu_{c}+e,$$

where Y is an n×1 observation vector of phenotypic value (n = 165, the number of segregants), X is a n×m design matrix relating observations to the mating-type locus and auxotrophic markers *LYS2* and *URA3* fixed effects β with β being a m×1 vector (m = 4, the four combinations of *LYS2* and *URA3* markers), $X_{q}$ is a n×q design matrix relating observations to the QTL parental allele fixed effects $\beta_{q}$ with $\beta_{q}$ being a q×1 vector (q = 4, the number of segregating parental alleles), Z is a n×n design matrix relating observations to the n×1 vector of polygenic additive random effects u, with $u∼N\left(0,A\sigma_{a}^{2}\;\right),$ and e is the n×1 vector of residual effects with $e∼N\left(0,I\sigma_{e}^{2}\right).$ I is an identity matrix and *A* is a genomic-based kinship based on segregation information away from the chromosome to which the position tested belongs. Coefficients of matrix A were calculated between pairs of individuals as the proportion of shared parental alleles on the segregating sites from the remaining chromosomes. At each tested position k, the linear mixed effect model was estimated by REML using the R-ASReml package for R, and p-values for the QTL fixed effect obtained with the Wald test. Vectors of 99,900 p-values were then adjusted for the false discovery rate (FDR) using R function p.adjust implementing the method from Benjamini and Hochberg (1995) and significant associations were retained at adjusted *q*-values = 0.5. Initially, we used this high threshold as a mean to obtain a greater number of putative QTL to incorporate a larger number of candidate genes into our screen. QTL mapping results are shown in Table S3.

### RNA-seq data analysis

A total of 16 individuals with extreme phenotypes for ammonium consumption (eight segregants with high and eight with low consumption levels, Table S2) were fermented. Fermentations were carried out as previously described in duplicate for each individual for 24 hr. Cultures were harvested by centrifugation and cells were treated with 2 U of Zymolyase for 30 min at 37°. RNA was individually extracted for each segregant utilizing the E.Z.N.A. Total RNA Kit I (OMEGA) according to the supplier’s instructions. RNA samples were then treated with DNase I (Promega) to remove genomic DNA traces and total RNA was recovered using the GeneJET RNA Cleanup and Concentration Micro Kit (Thermo Scientific). RNA integrity was confirmed using a Fragment Analyzer. Later, four RNA pools were generated utilizing equal amounts (1 μg) of RNA per individual: two high levels of ammonium consumption (HLA) and two low levels of ammonium consumption (LLA). Each pool was generated from independent replicates. The RNA-seq libraries were constructed using the TruSeq RNA Sample Prep Kit v2 (Illumina). Briefly, mRNA from 1 μg of total RNA was enriched by mRNA purification magnetic beads. Enriched mRNA was eluted and fragmented at 94° for 5 min. The double-stranded cDNA was acquired by RT-PCR using the above fragmented mRNA, followed by end repair, single A base addition, and adapter index ligation. The ligation product was amplified by PCR. The size of the end product was ∼260 bp. The sequencing was conducted on a HiSequation 2500 (Illumina) in a single lane for the four samples. Reads are available in the Biosample Database Project #PRJNA379146 with accession codes SAMN06602320, SAMN06602321, SAMN06602322, and SAMN06602323 for HLA-1, HLA-2, LLA-1, and LLA-2 samples respectively.

Raw reads were first assessed for their quality using the FASTQC tool kit (http://www.bioinformatics.babraham.ac.uk/projects/fastqc/). Low quality reads were discarded using the Trimmomatic tool using default score settings and a phred score cut-off of 30 (http://www.usadellab.org/cms/?page=trimmomatic). RNA-seq reads were then aligned to the S288c reference [*S. cerevisiae* genome obtained on 03/03/2016, from the *Saccharomyces* Genome Database (SGD), FTP SITE: http://downloads.yeastgenome.org/sequence/S288C_reference/genome_releases/ corresponding to a stable release from January 2015] utilizing Tophat with default score settings (Trapnell *et al.* 2009). BAM files were sorted and indexed using Samtools with default score settings (Li *et al.* 2009). Alignments were then processed and gene counts were attained utilizing HTSeq (Anders *et al.* 2015) with the no-stranded and –gene counts configuration from the S288c gff file. Differential expression was assessed with DESeq utilizing the nbinomTest function (Anders and Huber 2010). The results obtained from the DESeq analysis provided differentially expressed genes between pools with FDR < 0.05. FDR was estimated utilizing the default Benjamin–Hochberg correction (Benjamini and Hochberg 1995).

SNP frequencies were estimated by initially extracting SNP data from the four pools (two HLA and two LLA) utilizing the mpileup tool from Samtools with –u and –f options together with bcftools view –vcg options to export and obtain a .vcf file. Only high quality SNPs (at least 10 reads for the reference and alternative nucleotides) present in all four samples were considered. For this, we utilized the intersectbed tool from Bedtools filtering out SNPs absent in at least one of the pools (Quinlan and Hall 2010). Differential SNP frequency was estimated through a Fisher test using R and corrected using the *q*-value package with default options (Storey and Tibshirani 2003; Bass *et al.* 2015). A FDR < 0.001 was considered in order to obtain < 10 false positive results.

### Kyoto Encyclopedia of Genes and Genomes (KEGG) pathways enrichment

Differentially expressed genes and SNPs exhibiting significant frequency differences between pools were used in the DAVID Bioinformatics Resource (Huang da *et al.* 2009) to test for a significant enrichment of genes in pathways in the KEGG. FDR was estimated utilizing the default option (Benjamini and Hochberg 1995). We selected categories with a significant overrepresentation utilizing a FDR < 0.05%.

### Candidate gene validation

Genomic regions comprising 15 kb up- and downstream for selected QTL were examined in the SGD) for candidate genes. The sequences of the candidate genes were compared between strains utilizing SIFT analysis (Bergstrom *et al.* 2014). To validate the presence of a QTL, we performed a reciprocal hemizygosity assay (Steinmetz *et al.* 2002) (Figure S1). The gene *URA3* (essential for pyrimidine biosynthesis) previously deleted in the parental strains (Cubillos *et al.* 2009) was used as a selectable marker with some modifications. Briefly, we used haploid versions of the corresponding parental strains with extreme phenotypes for the QTL region to delete each target gene and construct all possible combinations of single deletions. Next, mutated parental strains were crossed to generate the reciprocal hemizygote strains and selected in double drugs plates (50 mg/ml Hygromycin B and 100 mg/ml Nourseothricin). The diploid hybrid strains were confirmed by *MAT* locus PCR (Huxley *et al.* 1990) and the deletions of the target genes were confirmed by PCR using the primers pairs A1/S8 or A4/S5 (Salinas *et al.* 2012). Nitrogen assimilation profiles between strains were compared using a classical Student’s *t*-test (p-value < 0.05). Total amino acid differences were compared through a paired *t*-test.

### Data availability

As stated above, genotype data can be obtained from Table S1. These genetic marker data were already published before in Cubillos *et al.* (2013). Phenotype data are available in Table S2. Gene transcript data can be obtained from the Biosample Database Project #PRJNA379146 with accession codes SAMN06602320, SAMN06602321, SAMN06602322, and SAMN06602323 for HLA-1, HLA-2, LLA-1, and LLA-2 samples, respectively. 

## Results

### Identification of genomic regions underlying nitrogen consumption differences in SGRP-4X

**Figure 1:**
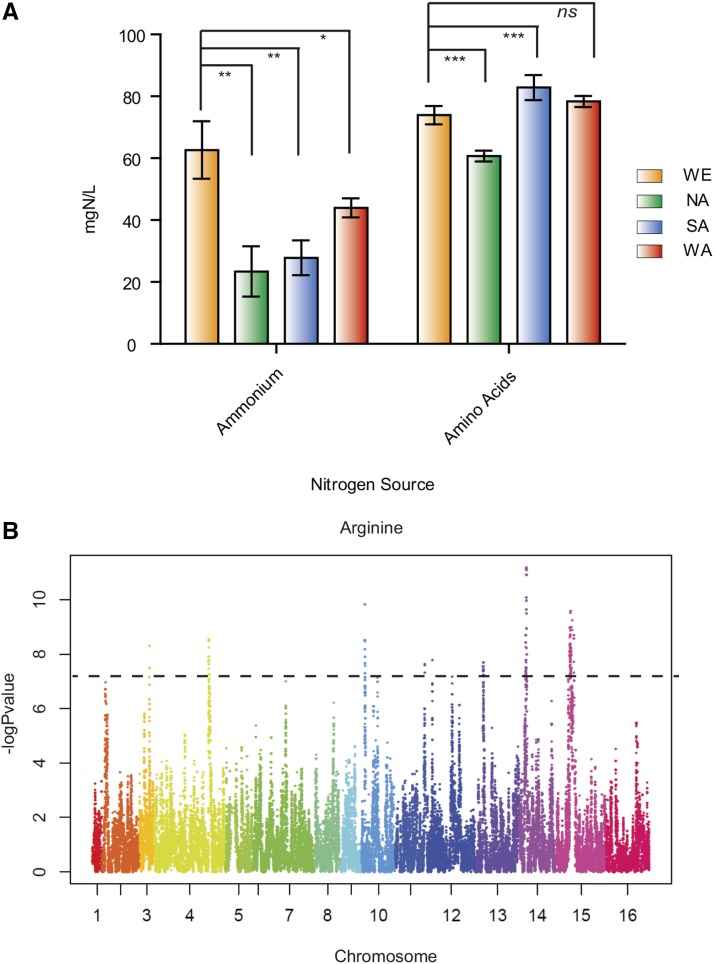
QTL Mapping in SGRP-4X. (A) Ammonium and amino acid total YAN consumption in parental strains. Student’s *t*-test was performed between the WE isolate and the other strains; * p-value < 0.05, ** p-value < 0.01, and *** p-value < 0.001. (B) Plot produced by QTL scan using Wald test for Arginine in the SGRP-4X population. The *y*-axis shows the p-value (–log10 scale) for the effect of a QTL along the chromosomes (*x*-axis), which are shown in different colors. Dotted line denotes cut-off FDR < 0.5. FDR, false discovery rate; ns, not-significant difference; QTL, quantitative trait loci; WE, Wine/European; YAN, assimilable nitrogen.

In order to obtain broader evidence of the genetic basis underlying nitrogen assimilation differences in *S. cerevisiae*, we selected a multi-parent recombinant population for QTL mapping analysis: SGRP-4X (Cubillos *et al.* 2013). The four parental founders of this population are representative of the NA (YPS128), SA (Y12), WE (DBVPG6765), and WA (DBVPG6044) clusters (Liti *et al.* 2009). Initially, we inoculated 1 × 10^6^ cells/ml in 12 ml of synthetic wine must (SM300) and characterized nitrogen consumption preferences in all founder strains after 6 d of fermentation. The WE isolate was able to consume greater levels of ammonium compared to any of the other strains (p-value < 0.05, *t*-test), as we have previously described (Jara *et al.* 2014). On the other hand, SA and WA isolates were able to efficiently consume amino acids rather than ammonium (Figure 1A), while the NA isolate consumed the lowest levels of amino acids and ammonium, representing the least adapted isolate to wine fermentation must. Based on this premise, a subset of 165 SGRP-4X fully sequenced recombinants were utilized to identify genetic variants responsible for nitrogen assimilation differences between strains (Cubillos *et al.* 2013). Thus, the whole population was phenotyped for consumption of 14 amino acids as well as ammonium (see *Materials and Methods*). Transgression levels were found to be relatively low, with the exception of serine and histidine, with 23.6 and 27.2%, respectively. Most transgressions were positive transgressive segregants (Table S2), suggesting that combinations resulting in an improved nitrogen assimilation profile are rare.

Large linkage blocks have historically been the main obstacle when analyzing complex traits at this experimental scale. A key reason for using the highly recombinant SGRP-4X population was to incorporate a greater genetic diversity and to distinguish and precisely map genes underlying a QTL within small linkage blocks compared to biparental F1 or F2 populations. Thus, to accurately identify genomic regions responsible for nitrogen consumption differences, we performed a QTL mapping strategy utilizing the high-resolution genetic map previously obtained for SGRP-4X (Cubillos *et al.* 2013) on the 15 phenotypes scored during the fermentation process. Each available marker across the genome (> 100,000 SNPs) was assessed for an association between the phenotype and parental allele fixed effects. We used a linear mixed model including correction for polygenic random effects and retained peaks with an empirical FDR < 0.5 (Cubillos *et al.* 2013). Overall, we were able to identify 29 QTL evenly distributed along the genome, aside from chromosomes I, V, VI, and VIII, which did not map for any QTL. Based on overlapping intervals, these QTL can be summarized into 26 major QTL regions (QTL1–QTL26), out of which three were present in more than a single nitrogen source (Table S4). In all cases, these pleiotropic QTL were found for two related amino acids. For example, QTL4 mapped for lysine (−logPvalue 12.26, FDR = 0.04) and arginine (−logPvalue 8.54, FDR = 0.14) traits, both amino acids with positively-charged side chains. Similarly, QTL10 mapped for phenylalanine (−logPvalue 14.39, FDR = 0.01) and methionine (−logPvalue 10.1, FDR = 0.3), two amino acids with hydrophobic side chains. These findings are in agreement with the phenotypic correlation between these couples exhibiting significant Spearman correlations (p < 0.05, Figure S2). On the other hand, arginine and isoleucine showed the greatest number of QTL with seven and six significant regions in each case, respectively (Figure 1B).

### Validation of candidates based on the QTL mapping approach

Based on our QTL mapping strategy, we looked for candidate genes for molecular validation. The majority of the QTL found in this study were mapped for arginine together with isoleucine; therefore, we focused on these amino acids for subsequent analysis (Figure 1 and Table S4). Thus, we examined all these QTL regions to select genes with a potential function in nitrogen metabolism. Three QTL were identified for arginine where candidate genes were identified. QTL21 mapped for isoleucine and peaks at chromosome XIV – 323 kb, which is 15 kb away from *ASI2*, a gene that encodes for an inner nuclear membrane protein and maintains the repressed state of gene expression in the absence of inducing amino acids (Forsberg *et al.* 2001). Similarly, QTL19 was identified in arginine peaks at chromosome XIV – 158 kb near *ALP1* and *LYP1*, two genes encoding for an arginine transporter and lysine permease, respectively. QTL8 mapped for arginine near *CPS1*, a vacuolar carboxypeptidase-S, shown to be under the influence of NCR and more particularly by *GLN3*. Expression of the vacuolar carboxypeptidase-S gene in *S. cerevisiae* is regulated by nutrient availability (Hofman-Bang 1999).

To determine the impact of the different allelic variants upon nitrogen consumption for these selected genes, we performed a functional analysis by hemizygotic strain comparison (Figure S1). For each gene, we selected the two parental strains for which the recombinant SGRP-4X segregants showed the most important differences in amino acid consumption for the QTL region implicated, particularly concerning arginine consumption for *LYP1*, *CPS1*, and *ALP1* genes, and isoleucine consumption for *ASI2*. In this way, the screening allows selection of the most appropriate parental strains for the cross and gene validation. The hybrid WE × SA was used to study *CPS1* for which segregants with the molecular markers originating from the WE background showed greater consumption levels for arginine compared to the SA strain. For the other three genes, *ALP1*, *LYP1*, and *ASI2*, we chose the WE × WA hybrid, for which the identification of molecular markers show a greater consumption of arginine, isoleucine, and tyrosine in the WE parental strain. Each cross included two allelic versions of the selected gene, one parent bearing an inactivated form and the other parent containing a functional form (Figure S1).

**Figure 2:**
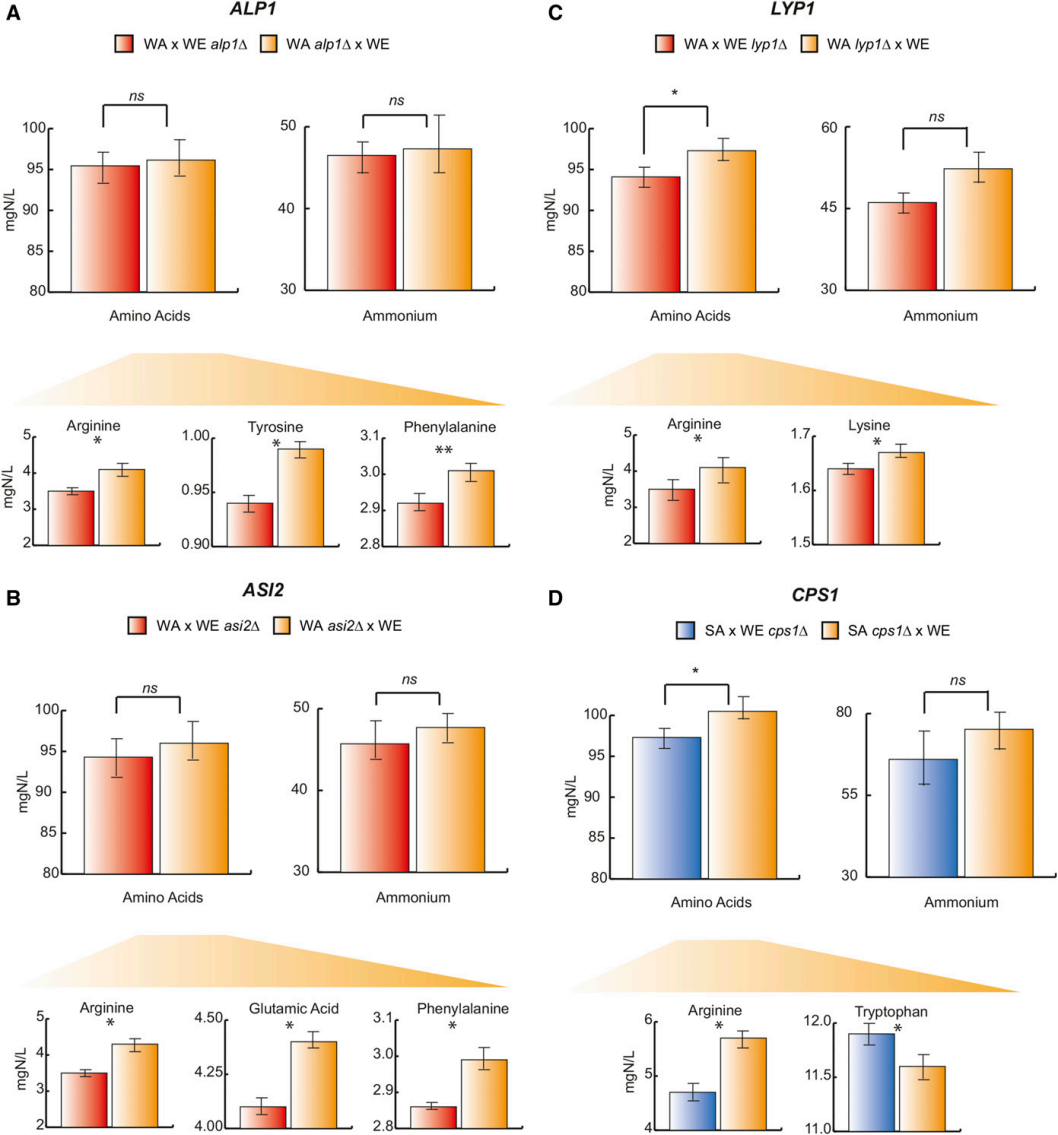
Reciprocal hemizygosity analysis on *ALP1*, *LYP1*, *ASI2*, and *CPS1* mapped through a QTL approach. The hybrid hemizygote strains consumption levels (mg/L) are shown. *, **, and *** represent a significant statistical difference between the hemizygote strains for the same gene using a *t*-test; * p-value < 0.05, ** p-value < 0.01, and *** p-value < 0.001. Reciprocal hemizygotes for (A) SA and WE *ALP1*, (B) *ASI2* WA and WE, (C) *LYP1* WA and WE, and (D) *CPS1* WA and WE. Δ/WE denotes hemizygotes carrying the WE allele, while XX/Δ denotes hemizygotes carrying the alternative allele. QTL, quantitative trait loci; SA, Sake; WA, West African; WE, Wine/European.

For the four genes tested, the comparison of residual amino acids revealed a statistically significant difference in YAN consumption for some amino acids (Table S5). Reciprocal hemizygosity analysis for *ALP1* showed that *ALP1^WE^* (reciprocal hemizygote carrying the WE allelic variant) conferred a higher consumption capacity for arginine, tyrosine, and phenylalanine, with a difference consumption of 17.1, 5.4, and 2.9%, respectively, between the two hemizygote constructions (p-value < 0.05, *t*-test, Figure 2A). These results are in agreement with the phenotype found in the SGRP-4X segregant population. The same findings apply for *ASI2*, with a greater consumption of glutamic acid, arginine, and phenylalanine sources (6.7, 17.3, and 4%, respectively) and marginally significant for isoleucine (p-value = 0.07, *t*-test), in the WE allelic variant compared to the WA background (Figure 2B). Concerning *LYP1*, the hybrid containing the WE allele provided a better arginine and lysine consumption capacity with a 37.5 and 1.8% difference between the two hemizygotes (Figure 2C). *CPS1* is the only gene for which the assessment of amino acid consumption between hemizygotes is different compared to the other genes. The allelic variant from the WE background showed significantly higher arginine consumption levels (equivalent to 19.5%), compared to the allele originating from SA; however, the *CPS1^SA^* reciprocal hemizygote showed greater tryptophan consumption levels (Figure 2D) equivalent to 2.6%, likely due to a compensation effect. Overall, these results demonstrate the role of these four genes in the YAN consumption phenotype and validate the power of the SGRP-4X multi-parent population to identify small-effect allelic variants.

### BSR-Seq identifies additional candidate genes

**Figure 3:**
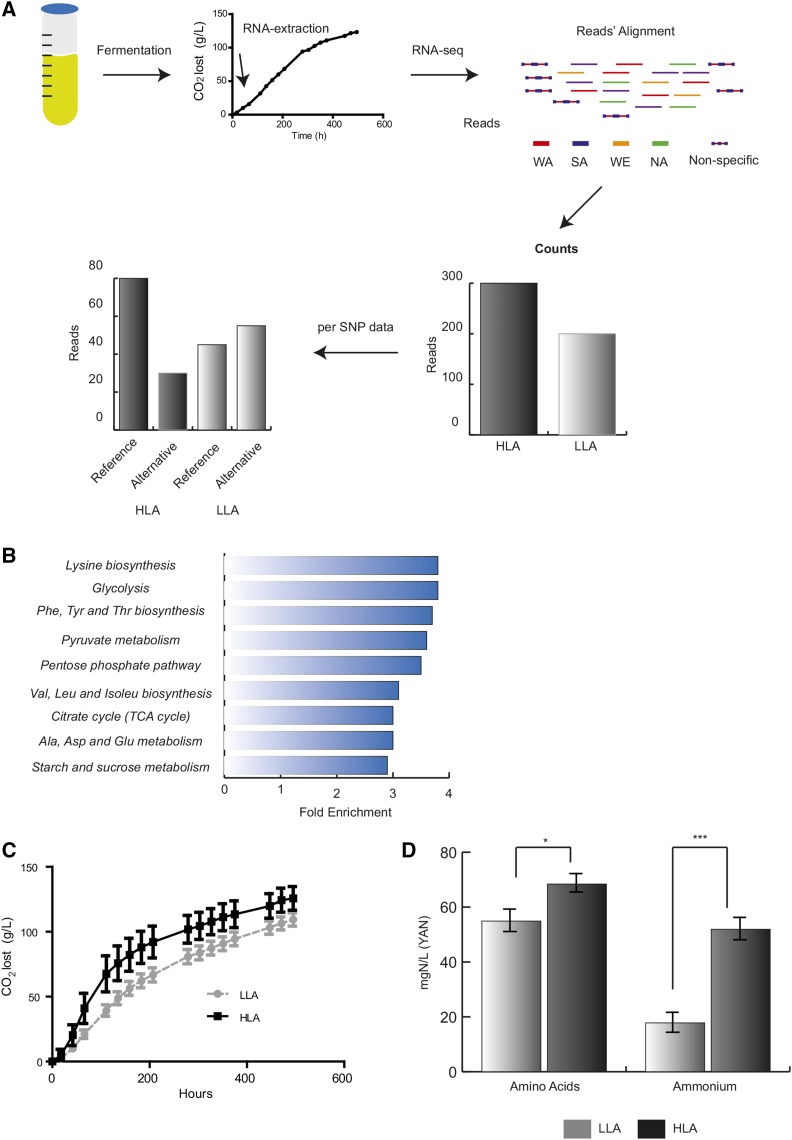
BSR-Seq experimental approach. (A) Experimental strategy followed to obtained mRNA counts and SNP frequencies from HLA and LLA pools. (B) Pathways enriched (FDR < 5%) in genes differently expressed between pools. (C) CO_2_ lost (g/L) in HLA (black) and LLA (dotted gray lines) pools. SDs are shown for each measured time-point. (D) Nitrogen consumption (mgN/L) in HLA (black) and LLA (gray) pools after 6 d of fermentation. * p-value < 0.05, ** p-value < 0.01, and *** p-value < 0.001. BSR-seq, Bulk segregant RNA-sequencing; FDR, false discovery rate; HLA, high levels of ammonium consumption; LLA, low levels of ammonium consumption; SNP, single nucleotide polymorphism.

Ammonium represents the main nitrogen source in wine must; therefore, in order to identify additional genetic variants underlying ammonium consumption differences, we utilized a BSR-Seq approach as a means to complement the QTL mapping strategy. We separately fermented eight individuals (in duplicate) that exhibited HLA consumption in the first round of fermentations and another eight with LLA (Figure 3 and Table S2). RNA was obtained after 24 hr of fermentation and four pools from each individual replicate were generated (two HLA and two LLA pools), which were sequenced through an Illumina HiSeq2500 platform (Figure 3A). We estimated differential expression between pools utilizing the DEseq package and found 725 genes differentially expressed at a 0.05 FDR (Table S6), with 491 genes expressed significantly higher in HLA compared to LLA and 234 vice versa.

**Figure 4:**
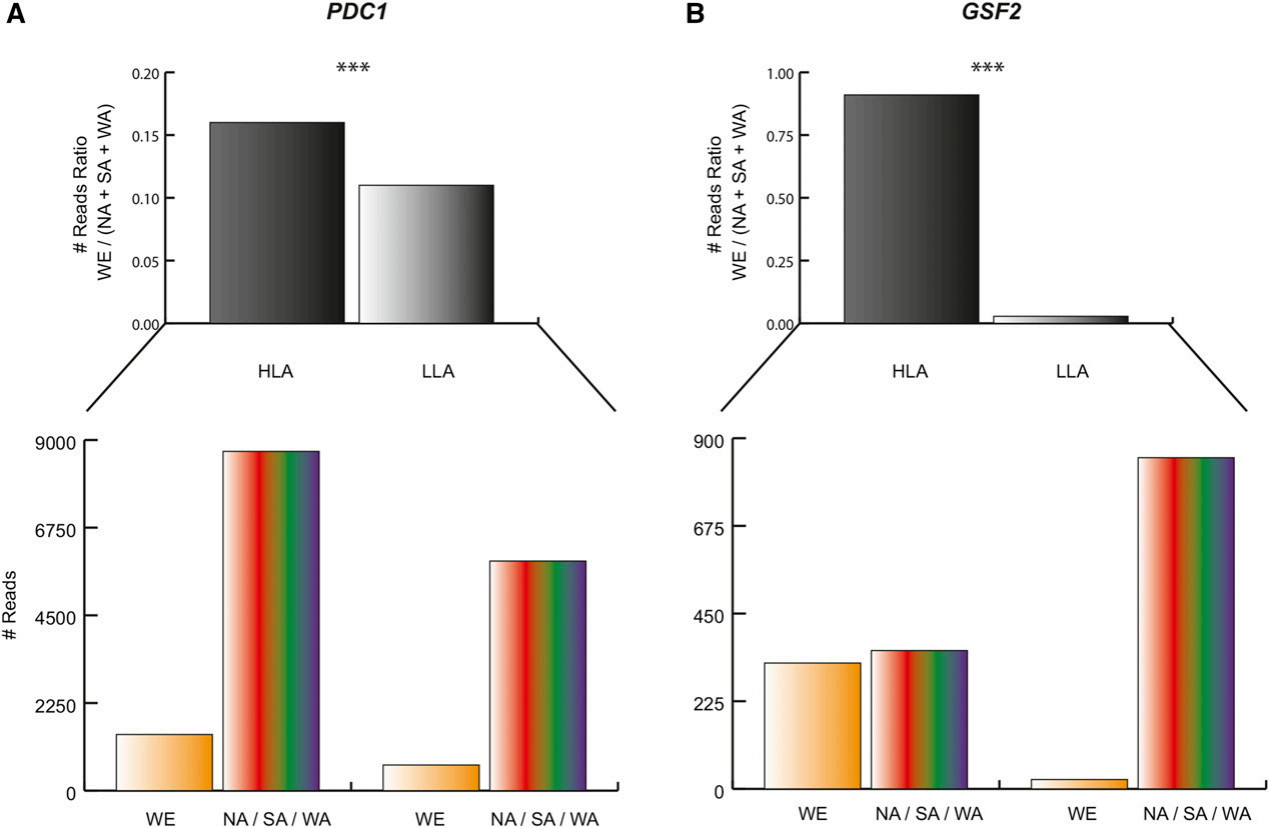
Read counts in genes differentially expressed between pools. Reads ratio [WE/ (NA + SA + WA)] for HLA (black) and LLA (gray) pools (upper panel) are shown. The number of reads per genotype, WE (orange) and NA + SA + WA (multi-color) bars, are depicted in the bottom panel. (A) SNP XII.232561 (chromosome XII – position 232561) located in *PDC1* and (B) SNP XIII. 179112 located in *GSF2*. HLA, high levels of ammonium consumption; LLA, low levels of ammonium consumption; NA, North American; SA, Sake; SNP, single nucleotide polymorphism; WA, West African; WE, Wine/European.

Subsequently, we utilized the KEGG database to determine what pathways could be enriched within this subset of genes. We found several implicated fermentation-related pathways associated with the differences found between pools, representing interesting candidates for gene validation (Figure 4B and Table S7A). Among others, we found carbon metabolism-related traits such as “glycolysis” and “citrate cycle (TCA cycle)” together with nitrogen metabolism traits such a: “phenylalanine, tyrosine, and tryptophan biosynthesis” and “alanine, aspartate, and glutamate metabolism,” with 10 and 12 genes, respectively, the majority of which were upregulated in the HLA pool. Indeed, these HLA segregants showed greater CO_2_ release levels (Figure 3C), finished the fermentation earlier, and consumed 5.4 times more ammonium than LLA segregants, demonstrating their greater fermentation capacity due to their higher consumption ability (Figure 3D).

Within each pathway, several genes emerged as causative candidates for the consumption differences between individuals. To help us elucidate causative genes and increase the power of our study, we initially focused the analysis on differentially expressed genes within nitrogen-related pathways such as: “phenylalanine, tyrosine, and tryptophan biosynthesis” together with the “alanine, aspartate, and glutamate metabolism” pathways and cross-referenced this data with the QTL mapping results. Moreover, to augment the likelihood of finding common regions, a p-value < 10^−6^ (−logPvalue > 6) was used for all phenotypes. Eight genes present in both datasets were found (Table S7B). Interestingly, *ARO1*, which encodes for a protein involved in chorismate biosynthesis (a precursor of aromatic amino acids), represents a good candidate since it emerged with a p-value < 10^−7^ in the QTL mapping approach for ammonium and is upregulated in the HLA pool with respect to the LLA pool (0.82 log_2_-fold change). Similarly, *HIS5*, which encodes for a histidinol-phosphate aminotransferase and is involved in the general control of amino acid biosynthesis, showed a p-value < 10^−9^ for isoleucine and is upregulated in HLA. Altogether, these RNA-seq results complement the QTL mapping approach and provide relevant genetic and biological insights toward the understanding of nitrogen source preferences and niche-ecological adaptation.

### Identification of SNPs highly enriched in pools of segregants

The BSR-seq approach enables the finding of differentially expressed genes between pools, but also allelic variants highly represented at the RNA level. These variants could be present because of allele enrichment in the pool of segregants or due to differences in expression levels, which consequently affect allele frequency distribution. In order to identify SNPs enriched within either pool that could be associated with the ammonium trait and find an additional set of genes that could complement the previous findings, we looked for single variants (utilizing samtools) by comparing the reads to the reference assembly (S288c). Overall, we identified 29,934 SNPs found in all four pools (HLA and LLA in duplicates), out of which only SNPs that were present in at least 10 reads within each pool were considered (Table S8). Next, we compared allele frequency ratios (#references/#alternative reads per nucleotide) between HLA and LLA pools through a Fisher exact test (Figure S3). A total of 2231 SNPs were found to be differentially represented between pools at a 0.001 FDR (< 2.2 false positives expected by chance). These SNPs belonged to 1460 genes and KEGG pathways analysis indicated that many are part of fermentation relevant traits (Table S9). Examples include: “Biosynthesis of secondary metabolites” (FDR = 0.0078) and “Biosynthesis of amino acids” (FDR = 0.01), extending the repertoire of functional variants for molecular validation. In this context, *PDC1*, a pyruvate decarboxylase that represents a key enzyme in alcoholic fermentation, was found within several of these pathways where the WE variant is highly represented in the HLA pool with respect to the LLA pool (Figure 4A, FDR = 10^−14^), and could partly explain its adaptation and affinity for ammonium consumption.

Consequently, we generated bulk expression frequency ratios between pools (HLA/LLA) and found 626 genes with ratios > 5 in either direction (335 HLA > LLA and 291 for LLA > HLA, respectively). Among those genes exhibiting extreme HLA/LLA ratios, we found *GSF2*, an integral membrane protein that may promote secretion of certain hexose transporters (Figure 4B). Moreover, the WE variant is highly represented in the HLA pool, suggesting a greater fermentation capacity of this allelic variant.

### Candidate validation derived from BSR-seq analysis

**Figure 5:**
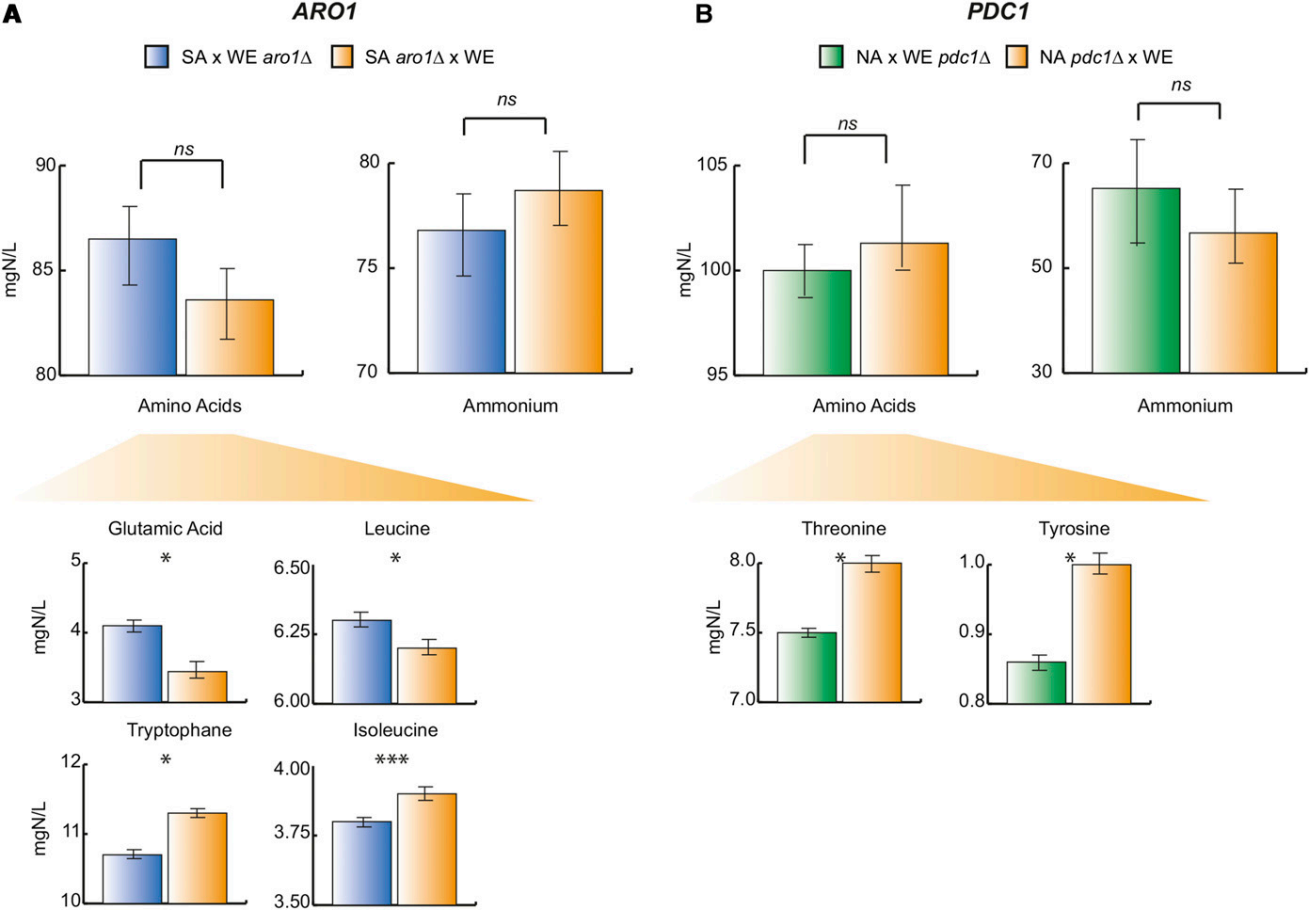
Reciprocal hemizygosity analysis on *PDC1* and *ARO1* genes mapped through BSR-seq approach. The hybrid hemizygote strains consumption levels (mg/L) are shown. *, **, and *** represent a significant statistical difference between the hemizygote strains for the same gene using a *t*-test; * p-value < 0.05, ** p-value < 0.01, and *** p-value < 0.001. Reciprocal hemizygotes for (A) NA and WE *PDC1*, and (B) *ARO1* WA and WE. Δ/WE denotes hemizygotes carrying the WE allele, while XX/Δ denotes hemizygotes carrying the alternative allele. BSR-seq, Bulk segregant RNA-sequencing; NA, North American; WA, West African; WE, Wine/European.

Allelic variants that emerged from our BSR-seq analysis were evaluated through reciprocal hemizygosity. *ARO1*, which encodes for a multifunctional enzyme involved in amino acid metabolism was found within a marginally significant QTL for ammonium at position IV.713 (−logPvalue = 6.94) and differentially expressed between pools (Table S6), where the SA allele was overrepresented in the LLA pool. Thus, we chose to use the WE × SA cross since the WE genotype was enriched in those SGRP-4X segregants exhibiting high ammonium consumption levels. Molecular validation in reciprocal hemizygotes demonstrated phenotypic differences for four amino acids (glutamic acid, tryptophan, leucine, and isoleucine) between allelic variants. Interestingly, glutamic acid and leucine were highly consumed by *ARO1^SA^*, while the aromatic amino acid tryptophan and the nonpolar isoleucine were preferentially assimilated by *ARO1^WE^*, in agreement with the “phenylalanine, tyrosine, and tryptophan biosynthesis” KEGG pathway findings (Figure 5A and Table S7). In contrast, we did not find differences for ammonium ion consumption, suggesting that *ARO1* might not be a direct causal factor for differences for this nitrogen source, but rather an intermediate with consequences upon the consumption of other sources.

Similarly, *PDC1*, a pyruvate decarboxylase, was chosen based on allelic frequency differences between HLA and LLA pools and its role in several overrepresented pathways (Table S9). In this case, the WE × NA hybrid was evaluated given the high frequency of the WE allele in the HLA pool. Reciprocal hemizygotes for *PDC1* showed statistically significant differences for tryptophan and tyrosine (p-value < 0.05, *t*-test), but not for ammonium as might have been expected (Figure 5B). In this case, *PDC1^WE^* showed a 6.2 and 13.1% greater consumption level for tryptophan and tyrosine, respectively.

## Discussion

The utilization of biparental crosses has been the main strategy utilized for QTL mapping and the identification of regions underlying complex traits, mostly because the limited number of sequenced strains has impeded other approaches (for example genome-wide association studies) (Liti and Louis 2012; Cubillos 2016). However, in most cases, mapped regions are limited to a few hotspots, such as: *HAP1*, *MKT1*, *IRA1*, and *IRA2* (Ehrenreich *et al.* 2009; Wang and Kruglyak 2014; Breunig *et al.* 2014). This is likely due to the utilization of a small set of biparental crosses between laboratory and wine domesticated strains, where a few polymorphisms have significant effects upon a wide set of phenotypes. In this context, the need for a greater panel of strains to understand the ecology, biology, genetics, and evolution of *S. cerevisiae* is vital.

To overcome the power limitation of genetically restricted biparental crosses, we previously reported the generation of SGRP-4X. This *S. cerevisiae* mapping population was obtained by outcrossing four founders representative of the main lineages for 12 generations (Cubillos *et al.* 2013; Burke *et al.* 2014), and contains 165 segregants with fine-grained mosaic genomes that have been sequenced for linkage mapping studies. We took advantage of the enormous sequence diversity of this population to unveil a greater number of genetic variants underlying nitrogen consumption differences between wild strains from different origins. Nitrogen consumption traits are important since: (i) nitrogen is an essential nutrient for any kind of fermentation, (ii) consumption profiles are genetically determined at the beginning of the fermentation process, and (iii) preferences are generally strain-dependent (Fleet 2003, 2008). Previous studies utilizing the four parental strains used in this study (and others) clearly demonstrate that natural strains vary greatly in their capacity to use nitrogen sources, likely due to differences in nitrogen metabolism, pathways, and more precisely in Ssy1-Ptr3-Ssy5 (SPS) and NCR systems between strains (Ibstedt *et al.* 2015; Crepin *et al.* 2012). These differences could be explained as an adaptive response to a specific nitrogen environment, particularly for the strains that have been isolated from a wide range of conditions (Liti *et al.* 2009).

In this study, we were able to extend the number of reported variants known to influence nitrogen consumption and specifically demonstrate that allelic variants in four candidate genes, mapped within three QTL for arginine, had an impact on YAN consumption performances (Figure 4). Our findings demonstrate that all tested genes have a mild, but significant effect on nitrogen assimilation. This can be explained by the extreme complexity of this population, with many allelic variants from different genetic backgrounds interacting within a single individual, buffering large effect alleles, and minimizing their impact upon phenotypes. Classical QTL mapping studies in yeast using two-parent crosses identify a few large effect loci per trait, missing small effect QTL (Bloom *et al.* 2015), complex gene–gene interactions (Ono *et al.* 2017), and a great fraction of pathways’ response programs (Gutin *et al.* 2015), which altogether shape an individual’s phenotype. Our results validate the role of several genes in arginine consumption and their likely allele–allele interactions with a functional compensation, difficult to detect in quantitative genetic and genomics studies utilizing two-parent crosses.

Interestingly, ammonium ion consumption was unaffected by the inactivation of the four alleles (Table S5), likely explained by the fact that ammonium ions are transported by three specific MEP permeases, namely Mep1p, Mep2p, and Mep3p (Marini *et al.* 1994, 1997), which were not mapped in our study. For three of the four genes, allelic variants with a positive effect on amino acid consumption belong to the WE genetic background, in agreement with the fact that this parental strain presents the highest YAN consumption and demonstrating that it likely possesses a selective advantage for oenological conditions. In this context, the WE background may have evolved a series of mechanisms and pathways to enforce the consumption capacity of overrepresented nitrogen sources (*i.e.*, ammonium, glutamine, and arginine) within wine must. Indeed, the BSR-Seq strategy demonstrated that carbon source assimilation pathways and nitrogen metabolism were significantly overrepresented in WE allelic variants and differentially expressed compared to other allelic backgrounds, demonstrating the underlying molecular mechanisms behind the greater fermentation profile in HLA segregants (Table S7 and Table S9). Differences in assimilation profiles and adaptation mechanisms could be driven by the availability of nitrogen sources within each strain’s niche. These key elements interact to determine ecological relationships between nutrient sources and strains’ adaptation capacity. In this context, a series of genomic changes have been associated with niche adaptation in wine yeast strains, such as *SSU1* (a sulfite-nitrite pump) translocations related to sulfite resistance and copy number variation of *CUP-1* (encoding a copper-binding metallothionein) implicated in elevated copper tolerance (Warringer *et al.* 2011; Marsit and Dequin 2015).

The comparison of the RNA-seq results and cross information with the QTL mapping strategy provided a set of genes enriched for metabolic pathways that could explain how strains were able to evolve and adapt to their preferred nitrogen sources and partly explain ecological differentiation. In particular, through RNA-seq, we were able to identify *PDC1* and *ARO1* as candidate genes. *PDC1* is a pyruvate decarboxylase Kellermann *et al.* (1986). This enzyme has a key role in the glycolytic pathway, which is essential for directing the glucose flux to ethanol production (Williamson *et al.* 1980). Coupled with two other decarboxylase proteins, Pdc5p and Pdc6p, Pdc1p contributes to the catabolism of branched amino acids (isoleucine and valine) and aromatic amino acids (phenylalanine and tryptophan) (Dickinson *et al.* 2003). In our study, we found assimilation differences between *PDC1* allelic variants for tyrosine (Table S5). Likewise, *ARO1* was found to underlie differences for several amino acids (Figure 5A). This gene encodes for an enzyme involved in the biosynthesis of aromatic amino acid precursors playing a central role in the superpathway of phenylalanine, tyrosine, and tryptophan biosynthesis (Duncan *et al.* 1987). Most of the genes encoding for the aromatic amino acid biosynthetic enzymes are regulated by the transcriptional activator Gcn4p which is a key player of the TOR pathway. Gcn4p participates in the regulation of the metabolic pathway GAAC (Hinnebusch and Fink 1983) and is potentially involved in the regulation of the NCR pathway (Tesniere *et al.* 2015). Besides its association with the TOR pathway, it was demonstrated that mutations within *ARO1* can cause the accumulation of intermediate metabolites (Lucchini *et al.* 1978) and disrupt the biosynthesis of certain amino acids. This disruption of the intracellular nitrogen pool could have repercussions on TOR sensing and signaling.

The QTL mapping strategy provided another set of genes that are also related to the TOR, SPS, and NCR pathways. *ASI2* is involved in the SPS signaling pathway through the transcription factor Stp1p (Zargari *et al.* 2007). Asi2p acts in a multi-protein complex together with Asi1p and Asi3p to ensure the control of the transcription factors Stp1p and Stp2p, regulating the stability of the SPS sensor system (Omnus and Ljungdahl 2014). In *S. cerevisiae*, the SPS signaling pathway enables cells to respond to the presence of extracellular amino acids and induce their uptake rate (Ljungdahl and Daignan-Fornier 2012). It is well known that the SPS system is upregulated at the beginning of the fermentation process (Ljungdahl 2009), suggesting that variation in Asi2p could modulate the SPS activity, resulting in gene expression differences associated with this system and impacting amino acid assimilation. On the other hand, *LYP1* encodes for a lysine permease whose activity is regulated by the SPS pathway. Lysine is an important amino acid for yeast nitrogen metabolism and fermentation performance (Lei *et al.* 2013), and the identification of allelic variants of *LYP1* supports the hypothesis of variation in SPS activity between parental strains. Our findings correlate well with this previous work, which demonstrated that lysine supplementation improves the expression of SPS-regulated genes, suggesting a fine modulation of the SPS system for the detection of extracellular amino acids. We were also able to validate the role of *ALP1*, encoding for an arginine transporter controlled by the NCR system. The nitrogen environment changes during fermentation, and yeast cells need to readapt their nitrogen metabolism (Tesniere *et al.* 2015). To achieve this, the NCR system is regulated by TOR activity, and previous studies have demonstrated that strains can have differences in TOR that impact their capacity to ferment (Brice *et al.* 2014a). Interestingly, *LYP1* and *ALP1* have high sequence homology and are adjacent (separated by 881 bp) (Sychrova and Chevallier 1994). However, they are controlled by two distinct nitrogen regulation mechanisms (SPS and NCR, respectively), demonstrating the architectural complexity of the phenotype. Similarly, we validated the role of *CPS1*, which encodes a permease involved in the degradation of proteins into amino acids, to provide nitrogen sources for the cell (Hofman-Bang 1999). Expression of *CPS1* is under the influence of NCR and more precisely the transcription factor Gln3p (Bordallo and Suarez-Rendueles 1993), which is in agreement with NCR being upregulated in the presence of arginine (Godard *et al.* 2007). Changes in *CPS1* activity can perturb the equilibrium of the nitrogen pool, impacting the TOR pathway and affecting NCR activity together with YAN consumption (Tesniere *et al.* 2015). In connection with these results, the pyruvate decarboxylase activity of Pdc1 is regulated by the protein phosphatase, Sit4p, which is also an important component of the TOR pathway. Sit4p is likewise involved in the regulation of the NCR system through Gln3. *PDC1* is a known candidate for regulation of fermentative activity (de Assis *et al.* 2013) and it is very likely that, in our study, variation in *PDC1* gene expression constitutes a marker determining the fermentative efficiency of a strain. This is coherent with the overexpression of *PDC1* in the WE parental strain (HLA pool), which is consistently the most suitable strain in the oenological fermentation environment. We can, thus, claim that, under oenological conditions, the WE strain shows the best capacity to use some of the available amino acids through the modulation of the TOR pathway sensing system.

### Conclusions

In this study, we identified a series of QTL and candidate genes through an integrative QTL-mapping–BSR-seq approach utilizing a complex AIL multi-parent population denominated SGRP-4X. The causal variants for *CPS1*, *LYP1*, *ALP1*, and *ASI2* are responsible for mild nitrogen assimilation differences for arginine, demonstrating how complex multi-parent populations can untangle small effect sources of natural variation. Our molecular validation demonstrates the role of several genes, which can confluence through multiple molecular mechanisms with significant effects on nitrogen assimilation, gene expression differences, and wine fermentation. In this context, these results support variations in nitrogen signaling pathways, and more precisely in SPS and NCR as the main systems responsible for differences in YAN consumption between strains from various origins. The SPS and NCR systems are key factors controlling nitrogen consumption and are highly conserved in *S. cerevisiae* (Crepin *et al.* 2012). In contrast, nitrogen metabolism regulation differs between strains (Gutierrez *et al.* 2013; Brice *et al.* 2014a; Jara *et al.* 2014), and variations in these systems could be linked to differences in the specific nitrogen environments causing ecological differentiation of these strains. Our results provide evidence of the molecular changes leading to niche adaptation with important implications for evolution and quantitative genomics in yeast.

## Supplementary Material

Supplemental material is available online at http://www.g3journal.org/lookup/suppl/doi:10.1534/g3.117.042127/-/DC1.
