# Supplementary figures and images for: Identification of Nitrogen Consumption Genetic Variants in Yeast Through QTL Mapping and Bulk Segregant RNA-Seq Analyses
Source: G3 (Bethesda). 2017 Jun 5;7(6):1693–705. doi: 10.1534/g3.117.042127 (PMC5473750; doi:10.1534/g3.117.042127)

## Phenotypic Correlation

A

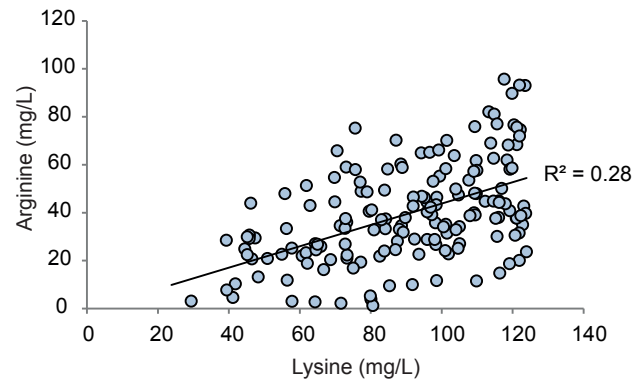

B

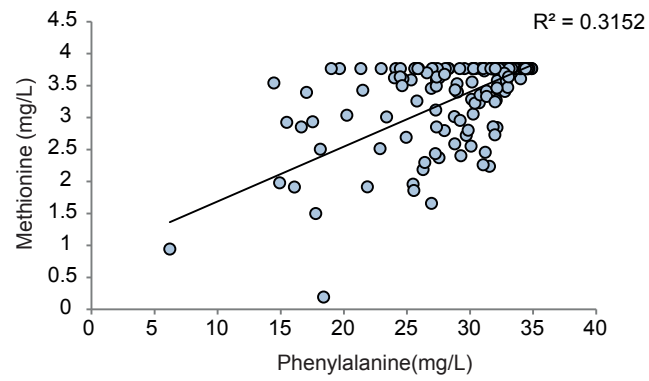

Supplement: Supplementary file 4 [file 1693File004.pdf]

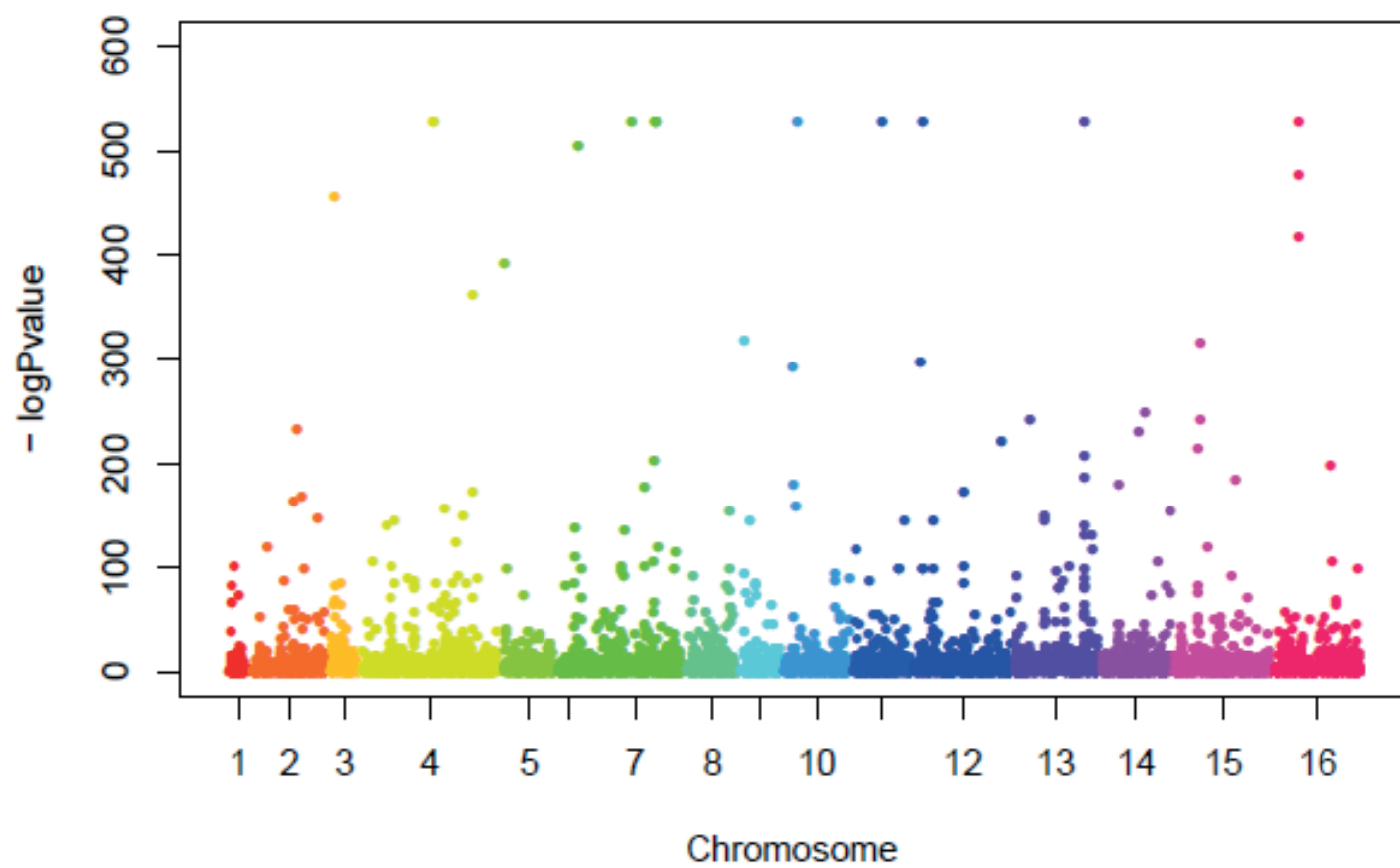

Figure S3

Supplement: Supplementary file 10 [file 1693File010.pdf]

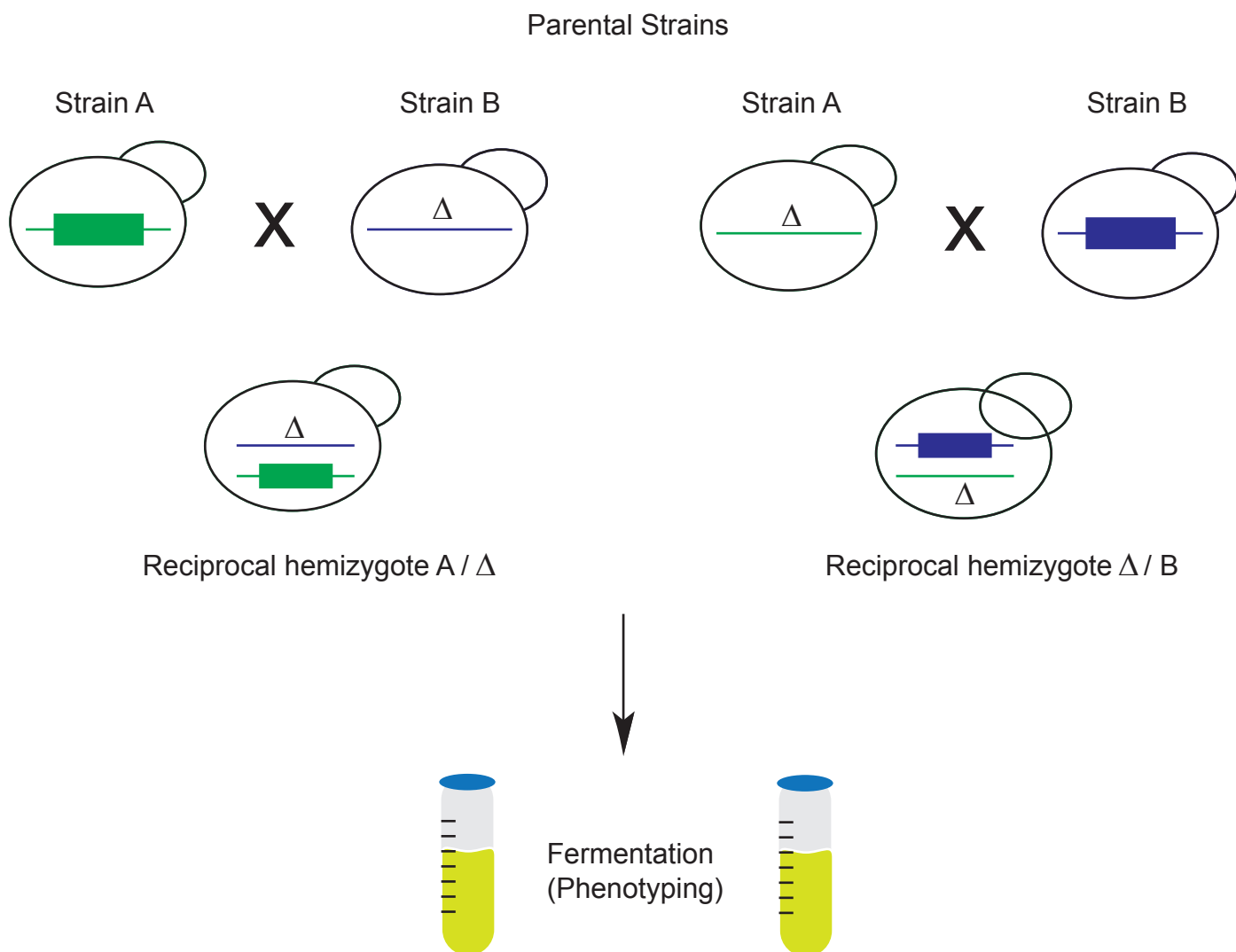

Figure S1

Supplement: Supplementary file 12 [file 1693File012.pdf]
