# Supplementary material for: Identification of Nitrogen Consumption Genetic Variants in Yeast Through QTL Mapping and Bulk Segregant RNA-Seq Analyses
Source: G3 (Bethesda). 2017 Jun 5;7(6):1693–705. doi: 10.1534/g3.117.042127 (PMC5473750; doi:10.1534/g3.117.042127)
Supplement: Supplementary file 8 [file 1693File008.docx]

1. ***Genetic Material***

The strains are publically available and have been previously released in Cubillos et al (2013). Strains can be requested to Francisco Cubillos ([francisco.cubillos.r@usach.cl](mailto:francisco.cubillos.r@usach.cl)) or Gianni Liti ([gianni.liti@ircan.fr](mailto:gianni.liti@ircan.fr)).

‘Viable spores with correct 2:2 segregations for the *MAT* locus and *ura3* and *lys2* auxotrophies were selected. We picked a total of 192 segregants (some from the same tetrad) from replicate 1 and stored them in glycerol stocks at 280’.

1. ***Raw Genotype file***

Supplemental Table S1. The code name of the segregant and the genotype NA = North America – YPS128, WE = Wine/European DBVPG6765, WA = West African DBVPG6044 and SA = Sake Y12 are shown. N/A denotes lack of genotype information.

1. ***Marker information file***

Supplemental Table S1. Markers are shown as ‘chromosome number. Position’. For example: II.23125 denotes chromosome II position 23125 bp.

1. ***Raw phenotype File***

Supplemental Table S2. Segregant's nitrogen consumption levels (mg/L) for 15 nitrogen sources.

1. **Description of Phenotypes**

Fermentations were carried out as previously described (Jara et al. 2014). Briefly, each individual was fermented in duplicates in synthetic wine must (SM300), prepared according to Rossignol *et al* (Rossignol et al. 2003). SM300 was supplemented with a final concentration of 300 mgN/L of assimilable nitrogen (YAN) corresponding to 120 mgN/L of ammonium and 180 mgN/L of a mixture of 19 amino acids (612.6 mg/L L-proline, 503.5 mg/L L-glutamine, 503.5 mg/L L-arginine monohydrochloride, 179.3 mg/L L-tryptophan, 145.3 mg/L L-alanine, 120.4 mg/L L-glutamic acid, 78.5 mg/L L-serine, 75.92 mg/L L-threonine, 48.4 mg/L L-leucine, 44.5 mg/L L-aspartic acid, 44.5 mg/L L-valine, 37.9 mg/L L-phenylalanine, 32.7 mg/L L-isoleucine, 50.0 mg/L L-histidine monohydrochloride monohydrate, 31.4 mg/L L-methionine, 18.3 mg/L L-tyrosine, 18.3 mg/L L-glycine, 17.0 mg/L L-lysine monohydrocloride, and 13.1 mg/L L-cysteine). The strains were initially grown under constant agitation in 10 ml of MS300 during 16 hours at 25ºC. Next, 1x10^6^ cells/mL were inoculated into 12 mL of MS300 (in 15 mL conical tubes) an incubated at 25ºC, with no agitations for six days, stage at which most nitrogen consumption differences can be observed (Martínez et al. 2013; Jara et al. 2014). After the six days, 12 mL of synthetic grape must (MSS300) were centrifuged at 9000x*g* for 10 minutes and the supernatant was collected. 20 uL of SM300 were injected in a Shimadzu Prominence HPLC equipment (Shimadzu, USA) using a Bio-Rad HPX –87H column according to Nissen et al. (Nissen et al. 1997). The concentration of each amino acid was measured using the HPLC analysis as previously described (Gomez-Alonso et al. 2007). The consumption of each nitrogen source was estimated as the difference between the initial and final amounts of each source before and after fermentation, respectively.

1. ***Results file***

Supplementary Table S3. Markers exhibiting at least one phenotype with –logPvalue > 6. Providing a table with roughly 100,000 markers and 15 traits would not be possible. Instead, we provide a table with the results for those markers showing high –logPvalues for at least a single phenotype. Table S6 depicts those genes differentially expressed between HLA and LLA pools utilising DESeq package and Table S8 the SNP counts in HLA and LLA pools and Fisher test results for differences between pools. The chromosome (chrm), position and gene where the SNP is located are depicted. The number of reads for the HLA-reference (HLA-Ref), HLA-SNP (HLA-Alt) and the equivalent for LLA, together with the respective ratios and p-values/FDR are shown. P-values were obtained through a Fisher t-test. Present genotypes are indicated with '1', while absent with '0'.
